# THE ROLE OF PRINT EXPOSURE IN SUPPORTING COGNITIVE ABILITY AMONG OLDER ADULTS

**DOI:** 10.1093/geroni/igz038.2415

**Published:** 2019-11-08

**Authors:** Xiaomei Liu, Elizabeth A L Stine-Morrow, and Giavanna S McCall

**Affiliations:** Beckman Institute and Educational Psychology, Urbana, Illinois, United States

## Abstract

Reading is an activity that has long been thought to have beneficial effects on cognitive ability, however, little is known about the effects of long-term reading engagement on cognition among older adults. Pre-test data are reported from an ongoing study of the effects of a literacy-based intervention on cognition among older adults. Participants (66.7% female) ranging from 60 to 79 years of age with education ranging from 11 to 19 years were administered a battery of measures, including print exposure checklists, which are objective indices for assessing long-term reading-related activities, as well as cognitive assessments. A composite measure of print exposure was based on the Author Recognition Test, Magazine Recognition Test, Non-fiction Author Recognition Test, and Fiction Recognition Test (alpha=.92). Cognition was assessed in a comprehensive battery, including measures of verbal ability, working memory, episodic memory, and verbal fluency (alpha=.83). Print exposure was marginally related to self-reported time spent reading (r=.27, p.4), suggesting criterion-related validity. Age-related declines were not evident for any of our cognitive measures in this sample, t(46)’s.8, and there was a modest increase in reading engagement with age (r=.36, p=.01). Controlling for verbal ability, print exposure had a significant positive influence on global cognition (standardized beta=0.38, p=.01); however, these effects were specific to episodic memory and verbal fluency, t(46)>2, p<.05. These preliminary findings are suggestive of selective cognitive benefits from continued literacy engagement in reading in later life.

